# Investigating the Hepatoprotective Properties of Mulberry Leaf Flavonoids against Oxidative Stress in HepG2 Cells

**DOI:** 10.3390/molecules29112597

**Published:** 2024-05-31

**Authors:** Qinhua Zheng, Ke Feng, Wenting Zhong, Weijian Tan, Sa Rengaowa, Wenzhong Hu

**Affiliations:** 1 College of Life Science, Zhuhai College of Science and Technology, Zhuhai 519041, China; 13726260359@163.com (Q.Z.); zwt_6249@163.com (W.Z.); wjtan20@mails.jlu.edu.com (W.T.); kuailexiaosa@sina.com (S.R.); 2 College of Life Science, Jilin University, Changchun 130012, China; 3 Faculty of Medicine, Macau University of Science and Technology, Macao 999078, China; fengkesky@163.com; 4 College of Life Science, Dalian Minzu University, Dalian 116600, China

**Keywords:** mulberry leaf flavonoids, oxidative stress, HepG2 cells, antioxidant activity

## Abstract

Oxidative stress significantly contributes to ageing and disease, with antioxidants holding promise in mitigating its effects. Functional foods rich in flavonoids offer a potential strategy to mitigate oxidative damage by free radicals. We investigated the protective effects of mulberry leaf flavonoids (MLF) against H_2_O_2_-induced oxidative damage in HepG2 cells. It assessed the inhibitory effect of MLF (62.5–500 μg/mL) on H_2_O_2_-induced oxidative damage by analyzing cellular morphology and oxidative stress markers, including ROS production, mitochondrial membrane potential, antioxidant enzyme levels, MDA, and apoptosis-related proteins. The results demonstrated that MLF prevented spiny cell formation triggered by 750 μM H_2_O_2_ and significantly reduced ROS levels, restored mitochondrial membrane potential, decreased lactate dehydrogenase and alanine transaminase leakage, and reduced MDA content induced by H_2_O_2_. MLF also modulated antioxidant enzymes and attenuated oxidative damage to HepG2 cell DNA, as confirmed by staining techniques. These findings indicate the potential of MLF as a hepatoprotective agent against oxidative damage in HepG2 cells.

## 1. Introduction

Reactive oxygen species (ROS) are oxidatively active intracellular oxygen–containing compounds in the body under oxidative stress. ROS can maintain the physiological homeostasis of body tissues and cells, but the presence of excess ROS in the body can disrupt its redox homeostasis and lead to peroxidative damage of body cells. Antioxidants can counteract the oxidative stress that causes many human diseases and eliminate ROS [1,2,3]. Therefore, it is important to find effective antioxidants for the prevention and treatment of related oxidative stress diseases. The number of free radicals maintains a dynamic balance, and a large accumulation of free radicals in the body can cause aging and disease. Flavonoids can prevent free radical damage in several ways, one of which is the direct scavenging of free radicals. Flavonoids are oxidized by free radicals, producing more stable and less reactive free radicals. In other words, flavonoids stabilize reactive oxygen species by reacting with the reactive compounds of free radicals. Due to the high activity of the hydroxyl groups of flavonoids, free radicals are inhibited and some flavonoids can directly scavenge superoxide, while others can scavenge highly reactive oxygen-derived free radicals called peroxynitrite. In in vitro studies, by scavenging free radicals, flavonoids can inhibit LDL oxidation, a behavior that protects LDL particles, and flavonoids may theoretically have a preventive effect on atherosclerosis [4,5]. Flavonoids, including catechins, are reported to be powerful antioxidants protecting the body from reactive oxygen species. Human cells and tissues are continuously threatened by damage from free radicals and reactive oxygen species generated during normal oxygen metabolism or induced by exogenous damage [6]. The mechanisms and sequence by which free radicals interfere with cellular function are not fully understood, but the most important is lipid peroxidation, which leads to cell membrane damage, and this cellular damage results in net charge transfer, altered osmotic pressure, resulting in swelling, and eventually cell death. Free radicals lead to inflammatory responses and tissue damage. Antioxidant defense mechanisms in the human body include not only enzymes such as superoxide dismutase, catalase, and glutathione peroxidase but also non-enzymatic counterparts such as glutathione, ascorbic acid and *α*-tocopherol [7,8].

*Morus* spp. is a deciduous tree belonging to the genus *Morus* in the family Moraceae and is widely distributed in tropical, subtropical, and temperate regions worldwide [9]. Mulberry leaves are the dried leaves of *Morus alba*, family Moraceae, and are used as herbal medicine in several East Asian countries including China, Japan, and Korea because of their various pharmacological effects. The chemical composition of mulberry leaves is complex and contains a variety of active ingredients such as flavonoids, alkaloids, phytosterols, γ-aminobutyric acid, and mulberry leaf polysaccharides; therefore, mulberry leaves have a wide range of biological activities and clinical utilization values. Mulberry leaves are considered a potentially important functional food due to their bioactive compounds, while their biological activity is related to the chemical composition, which includes flavonoids (anthocyanins, rutin, quercetin, and isoquercitrin), steroids, amino acids, polysaccharides, y-aminobutyric acid (GABA), vitamins and 1-deoxynojirimycin (DNJ) [10], molecules that can directly neutralize reactive oxygen species (ROS) and/or deactivate molecules with pro-oxidant capacity. Flavonoids are a large group of polyphenolic compounds found in fruits and vegetables. Mulberry leaves are known to be rich in flavonoids, including quercetin, rutin, isoquercitrin, anthocyanin 3-rutinoside, and anthocyanin 3-glucoside [11]. These compounds are known to have potential antioxidant properties and may play a role in the prevention of oxidative stress-related diseases.

H_2_O_2_ is considered a reactive oxygen species (ROS) because of its ability to generate highly reactive hydroxyl radicals by interacting with reactive transition metals [12,13], in addition, due to its non-ionized and low-charge state, H_2_O_2_ is able to diffuse through hydrophobic membranes and generate a series of free radicals. Free radicals and ROS, interact with lipids, DNA, and proteins, thus degrading proteins and promoting DNA strand breaks and damage to other genomic structures. These reactive substances also affect lipids and disrupt the integrity of polyunsaturated fatty acids, which in turn affects the homeostatic environment of cells [14]. Human hepatocellular carcinoma cells (HepG2) are typical model cells and are commonly used for in vitro antioxidant activity studies [15,16]. In this study, HepG2 cells were used to investigate the protective effect of MLF [15,16] on H_2_O_2_–mediated oxidative damage in HepG2 cells. By measuring the markers of oxidative stress, mitochondrial dysfunction and apoptosis (MDA, ALT, LDH, SOD, CAT, GSH–Px, ROS, JC–1, apoptosis rate) and cell morphology of HepG2 cells, the To investigate the antioxidant activity of MLF at the cellular level. This study aims to provide technical and theoretical guidance applying MLF in the development of functional ds and healthy foods.

## 2. Results

### 2.1. Effect of MLF on Survival of HepG2 Cells with H_2_O_2_–Mediated Oxidative Damage

**Figure 1:**
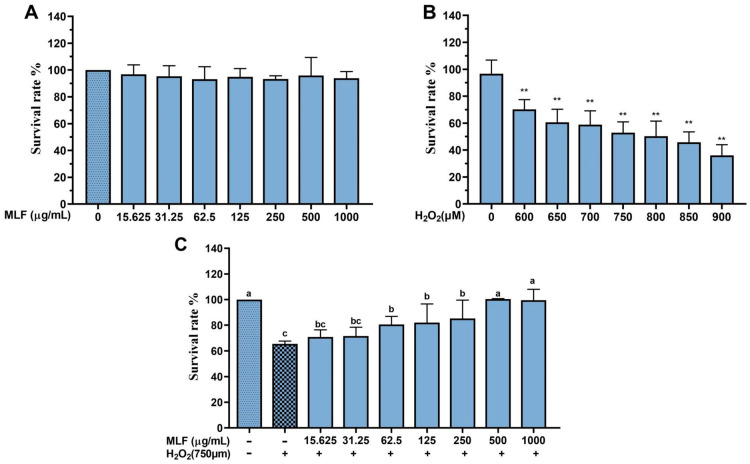
The survival rates of HepG2 cells treated with MLF (**A**), H_2_O_2_ (**B**), both MLF and H_2_O_2_ (**C**) at different concentration (+ represents addition; − represents without addition; ** *p* < 0.01, versus H_2_O_2_ group, *n* = 3).

H_2_O_2_ can easily cross the cell membrane to damage cells [17,18]. To investigate whether MLF can protect cells from H_2_O_2_–mediated oxidative damage, first, the effect of different concentrations of MLF on the survival rate of HepG2 cells was investigated. As shown in Figure 1A, the cell viability of HepG2 cells treated with MLF at concentrations ranging from 15.625 to 1000 μg/mL ranged from 93.18% to 96.74%, and the results indicated that there was no significant difference between the cell viability after MLF treatment and the blank control (*p* > 0.05), and the selected MLF concentrations were non-toxic to HepG2 cells. The following experiments were performed at these non-toxic concentrations.

As shown in Figure 1B, the survival rates of HepG2 cells were examined after 5 h of incubation in 600–900 μM H_2_O_2_. The results showed that the survival rate decreased significantly with the increase of H_2_O_2_ concentration in a dose-dependent manner. Among them, the survival rate of HepG2 cells decreased to 50.88% (** *p* < 0.01) when incubated in 750 μM H_2_O_2_, which was closest to the IC50; therefore, 750 μM H_2_O_2_ was used for the follow–up experiments to evaluate the protective effect of MLF on HepG2 cells.

HepG2 cells were incubated with MLF at concentrations of 13.625~1000 μg/mL for 24 h and then treated with H_2_O_2_ (750 μM) for 5 h. As shown in Figure 1C, the survival rate of HepG2 cells in the damage group was only 65.42 ± 2.25%, while the survival rate of HepG2 cells treated with different concentrations of MLF was significantly higher than that of the damage group (*p* < 0.05), i.e., MLF could significantly increase the survival rate of HepG2 cells, and the above results indicated that MLF could effectively reduce the oxidative damage induced by H_2_O_2_ in HepG2 cells. Therefore, 62.5, 125, 250, and 500 μg/mL MLF were selected for follow-up experiments to evaluate the protective effect of MLF on HepG2 cells.

### 2.2. Effect of MLF on the Apoptosis Rate of HepG2 Cells with H_2_O_2_–Mediated Oxidative Damage

**Figure 2:**
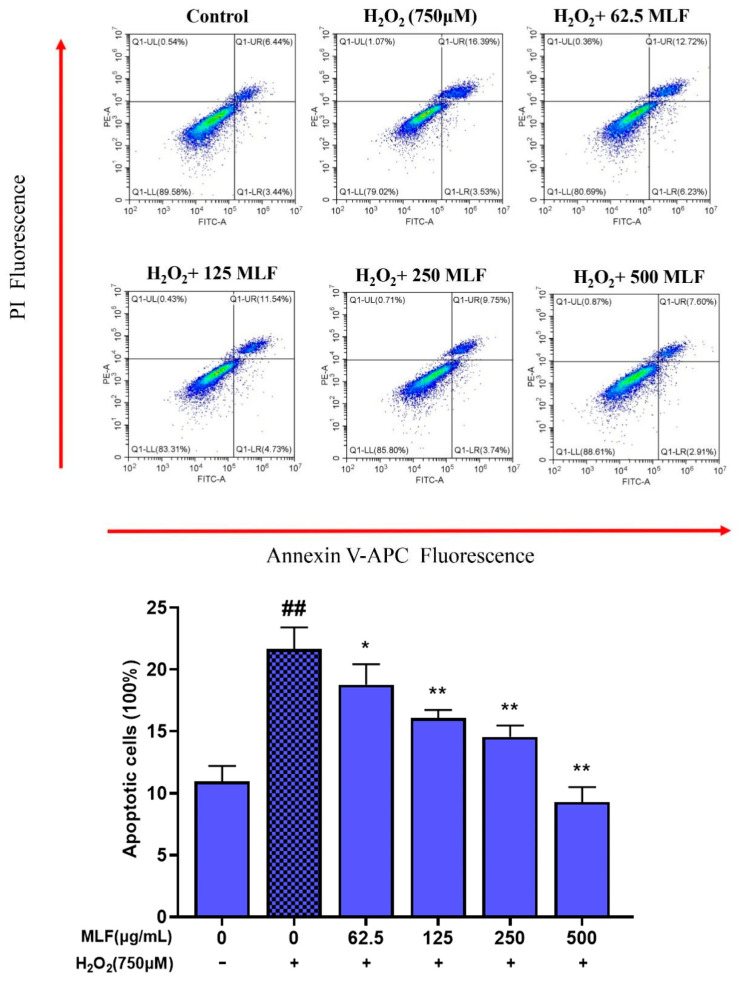
The apoptosis rates of HepG2 cells at different treatments (control, without addition of MLF and H_2_O_2_; damage addition of 750 μM H_2_O_2_; MLF protect, addition of MLF and 750 μM H_2_O_2_) (+ represents addition; − represents without addition; * *p* < 0.05, ** *p* < 0.01, versus H_2_O_2_ group, ## *p* < 0.01 versus control, *n* = 3).

Apoptosis plays an important role in many processes, and abnormal apoptosis is associated with many human diseases of many kinds, such as ischemic injury, neurodegenerative diseases, autoimmune diseases, and cancer [19]. Apoptosis assays are commonly used to evaluate the potential use of bioactive ingredients as food supplements or chemotherapeutic agents. The apoptosis rate of HepG2 cells uninjured by H_2_O_2_ oxidative stress was low at 9.88% (Figure 2). 5 h after oxidative stress injury by 750 μM H_2_O_2_, the apoptosis rate increased dramatically to 19.92%. In contrast, the apoptosis rate of HepG2 cells was significantly lower (*p* < 0.05) after pretreatment with 62.5~500 μg/mL MLF for 24 h followed by H_2_O_2_ treatment, with the lowest apoptosis rate of 10.51% for 500 μg/mL MLF. The above results indicated that MLF had a protective effect on H_2_O_2_–mediated oxidative stress injury in HepG2 cells, with the most significant protective effect of 500 μg/mL MLF.

### 2.3. Analysis of Hoechstand Trypan Blue Staining

**Figure 3:**
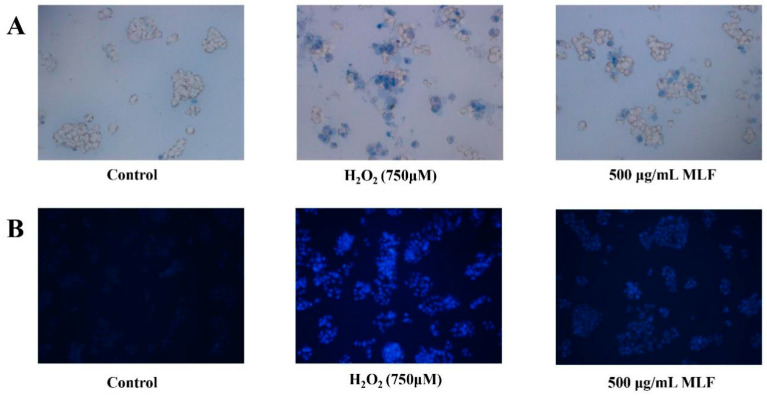
Chromatin images of HepG2 cells stained with (**A**) Hoechst 33342 and (**B**) trypan blue (Control, without the addition of MLF and H_2_O_2_; damage, the addition of 750 μM H_2_O_2_; MLF protect group, the addition of 500 μg/mL MLF and 750 μM H_2_O_2_).

The effect of MLF on cells treated with H_2_O_2_ was assessed using trypan blue and Hoechst 33342 staining. Trypan blue selectively stains dead cells, whereas Hoechst 33342 stains both living and dead cells. Therefore, the combined use of these stains allows for a comprehensive assessment of cellular viability and death. To investigate the protection mechanism of MLF against H_2_O_2_–mediated oxidative damage in HepG2 cells, Hoechst 33342 and trypan blue staining assay of HepG2 cells were performed. The chromatin images of HepG2 cells in the blank control group, the damage group and the MLF-protected group are shown in Figure 3. The control group exhibited a uniform light blue color and a few fluorescent spots. The damage group exhibited a large number of strong fluorescent spots. In contrast, the MLF-protected group showed weaker fluorescent spots and fewer fluorescent blue cells than the damaged group. Meanwhile, the results of trypan blue staining showed that the dead cells were stained blue by trypan blue, and the dead cells were significantly reduced after MLF treatment compared with the damage group. These phenomena suggest that MLF can play a protective role for HepG2 cells by attenuating the oxidative damage of HepG2 cell DNA.

### 2.4. Effects of MLF Pretreatment on ROS Level in HepG2 Cells

**Figure 4:**
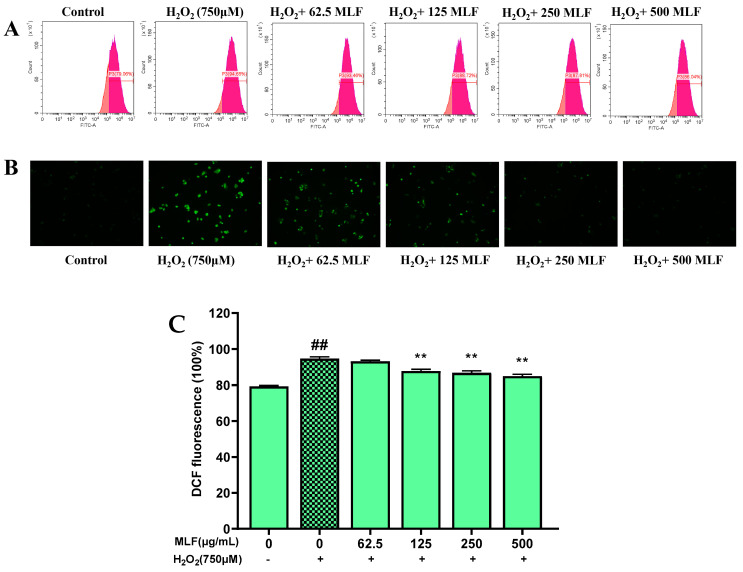
The ROS levels of HepG2 cells at different treatments (control, without the addition of MLF and H_2_O_2_; damage, the addition of H_2_O_2_; MLF protect, the addition of MLF and H_2_O_2_). Where (**A**) is the fluorescence intensity of ROS probe detected using flow cytometry, (**B**) is the fluorescence intensity of cellular ROS detected using fluorescence inverted microscopy, and (**C**) statistical of fluorescence intensity based on flow cytometry detection (+ represents addition; − represents without addition; ** *p* < 0.01, versus H_2_O_2_ group, ## *p* < 0.01 versus control, *n* = 3).

The protective effect of MLF against H_2_O_2_-induced oxidative stress was further investigated by measuring ROS levels in HepG2 cells by the DCFDA method. The fluorescent product dichlorofluorescein (DCF) is produced in cells by oxidation of DCFH_2_ by intracellular ROS (especially H_2_O_2_) [20]. Therefore, elevated levels of ROS indicate an increase in oxidative stress. The protective mechanism of antioxidants against free radical-induced apoptosis may be explained by their ability to scavenge ROS generation in the organism. As shown in Figure 4, the inhibitory effects of different concentrations of MLF on H_2_O_2_-induced ROS production in HepG2 cells were determined. The 750 μM H_2_O_2_ treatment of HepG2 cells for 5 h resulted in significantly higher ROS levels than the control (*p* < 0.05). MLF pretreatment significantly inhibited the increase in intracellular ROS levels (*p* < 0.05) in a dose-dependent. ROS levels were reduced by 8.61% in MLF-pretreated (500 μg/mL) cells compared to H_2_O_2_-treated HepG2 cells.

### 2.5. Effect of MLF Pretreatment on Biochemical Parameters in HepG2 Cells

To reveal the protective effect and antioxidant mechanism of MLF, the effects of MLF on lactate dehydrogenase (LDH) and glutamate transaminase (ALT) release, malondialdehyde (MDA) content and superoxide dismutase (SOD), glutathione peroxidase (GSH-Px)) and catalase (CAT) levels in H_2_O_2_-induced oxidative damage in HepG2 cells were investigated.

**Figure 5:**
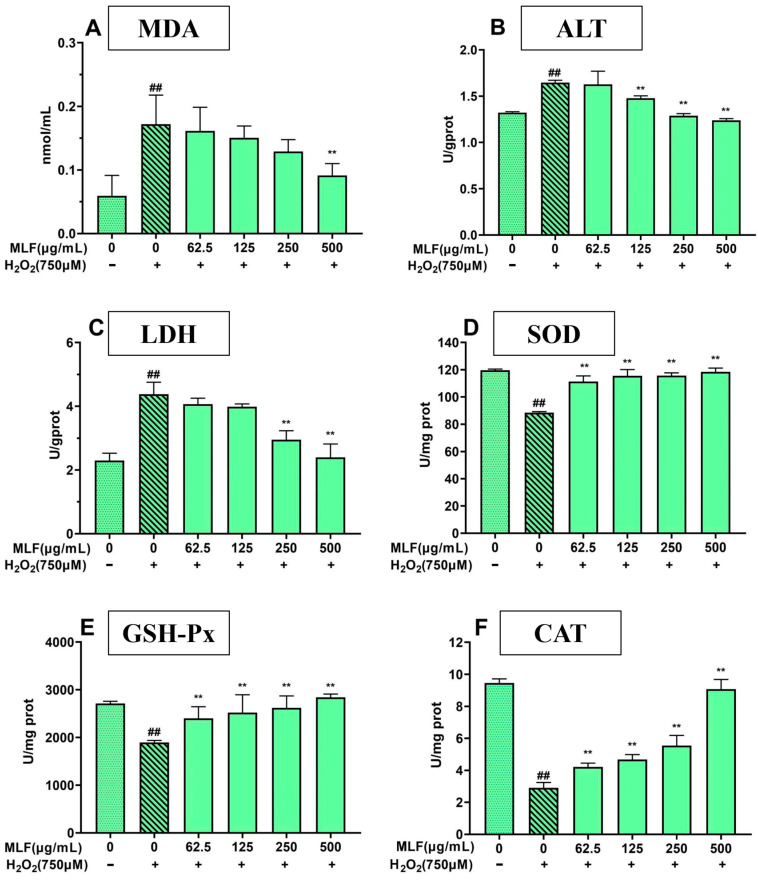
The MDA (**A**), ALT (**B**), LDH (**C**), SOD (**D**), GSH-Px (**E**)and CAT (**F**) activities of HepG2 cells at different treatments (control, without the addition of MLF and H_2_O_2_; damage, the addition of H_2_O_2_; MLF protect, the addition of MLF and H_2_O_2_) (+ represents addition; − represents without addition; ** *p* < 0.01, versus H_2_O_2_ group, ## *p* < 0.01 versus control, *n* = 3).

As shown in Figure 5, the MDA content of HepG2 cells after H_2_O_2_ treatment was significantly higher than that of the blank control cells (*p* < 0.01). In addition, LDH and ALT release were significantly higher, and the activities of three antioxidant enzymes, GSH-Px and CAT, were significantly lower (*p* < 0.01), which may be due to the severe membrane damage induced by H_2_O_2_ in HepG2 cells. HepG2 cells treated with MLF could significantly reduce the release of LDH and ALT in cells, and the above phenomena were significantly improved in a concentration-dependent manner, indicating that MLF can repair and protect cells from H_2_O_2_-induced oxidative damage, regulate the activity of antioxidant enzymes to resist oxidative damage caused by H_2_O_2_, and reduce the elevated MDA content and abnormal leakage of ALT and LDH caused by H_2_O_2_.

### 2.6. Effect of MLF Pretreatment on Mitochondrial Membrane Potential Levels in HepG2 Cells

**Figure 6:**
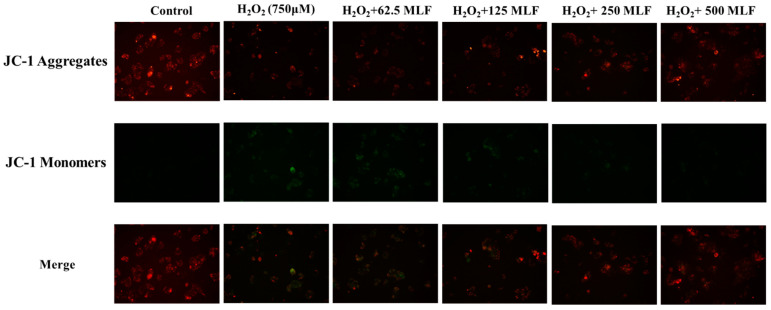
Effect of MLF pretreatment on mitochondrial membrane potential in injury models.

To date, mitochondrial dysfunction has been identified as a key organelle involved in apoptotic events, and the dynamic changes of mitochondria in H_2_O_2_–mediated oxidative stress experiments in HepG2 cells were assessed by fluorescence inverted microscopy. Analysis of the functional state of mitochondria in HepG2 cells without MLF treatment as shown in Figure 6 revealed that after 5 h of culture, the mitochondrial transmembrane potential was significantly reduced compared to that of healthy (control) HepG2 cells, highlighted by an increase in the total number of depolarized cells in the group. HepG2 cells treated with MLF eliminated the loss of mitochondrial membrane potential, indicating a significant decrease in total depolarized mitochondria-positive cells. The experimental results suggest that MLF protects cells from the H_2_O_2_-induced decrease in mitochondrial transmembrane potential in HepG2 cells.

### 2.7. Results of Apoptosis-Related Protein Assay in HepG2 Cells

**Figure 7:**
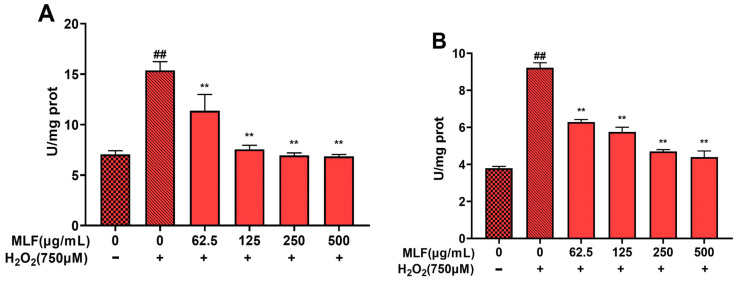
Quantitative analysis of MLF pretreatment on apoptosis in injury models, where (**A**) is the result of caspase-9 protein content measurement, and (**B**) is the result of caspase-3 protein content measurement (+ represents addition; − represents without addition; ** *p* < 0.01, versus H_2_O_2_ group, ## *p* < 0.01 versus control, *n* = 3).

Apoptosis involves the activation, expression and regulation of a series of proteins. Among them, caspase family proteins play an important role in signal transduction and can be activated by death receptors and mitochondrial pathways [20,21]. As seen in Figure 7, H_2_O_2_ treatment significantly upregulated the expression of caspase-3 and caspase-9 proteins in HepG2 cells (*p* < 0.01), and MLF (62.5–500 μg/mL) pretreatment of HepG2 cells significantly decreased the expression of caspase-3 and caspase-9, and the protein expression levels were dose dependent.

### 2.8. Observation of Cell Morphology

**Figure 8:**
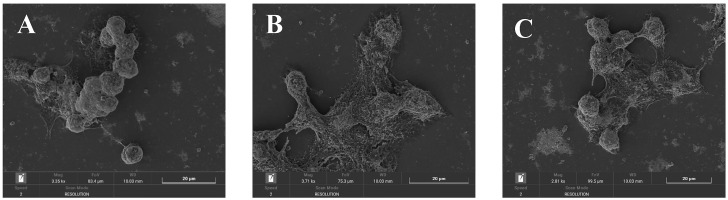
SEM images of HepG2 cells at different treatments. Control group (**A**), without addition of MLF and H_2_O_2_; Damage group (**B**), addition of 750 μM H_2_O_2_; MLF protect group (**C**), addition of MLF (500 μg/mL) and 750 μM H_2_O_2_.

SEM images of HepG2 cells cultured in normal medium for 24 h (control group), cultured in normal medium for 24 h, then treated with 750 μM H_2_O_2_ for 5 h (damage group), pretreated with 500 μg/mL MLF for 24 h, then treated with 750 μM H_2_O_2_ for 5 h (MLF-protected) are shown in Figure 8. H_2_O_2_–mediated oxidative damage injury significantly altered the surface structure of HepG2 cells. The control group HepG2 cells were elongated and filamentous microvilli were clearly visible (Figure 8A). After oxidative stress from H_2_O_2_–damage, the cell volume increased dramatically and the cell surface appeared to rise or collapse (Figure 8B). Under the protection of MLF, some intact cells were preserved, but some damaged cells with increased volume and collapsed surfaces were still present (Figure 8C). These images provide clear experimental evidence that MLF protects HepG2 cells from H_2_O_2_–mediated oxidative damage.

## 3. Materials and Methods

### 3.1. Materials and Chemicals

Mulberry leaf flavonoids (MLF) [13] are provided by the laboratory of the School of Pharmacy and Food Science, Zhuhai Institute of Science and Technology. Cellulase(CAS: 9012-54-8) and pectinase(CAS: 9032-75-1) were purchased from Shanghai Yuanye Biotechnology Co., Ltd. (Shanghai, China) and macroporous resin D101 was purchased from Donghong Chemical Co. Special Fetal Bovine Serum (FBS) was purchased from Procell Life Science & Technology Co., Ltd. (Wuhan, China). Phosphate–buffered saline (PBS, pH 7.4), DMEM nutrient solution and penicillin-streptomycin double antibody were obtained from Darmstadt, Germany. H_2_O_2_ was purchased from Sangon Biotech (Shanghai, China) Co., Ltd. Trypsin was purchased from Solaibao Biotechnology Co., Ltd. (Beijing, China) Reactive oxygen detection (ROS) kit, Cell Counting Kit-8, Hoechst 33342 dye solution and 5,58,6,68–tetraethyl–benzimidazol–carbocyanine iodide ((JC-1) were bought from Beyotime Biotechnology Co. The high-efficiency RIPA lysis solution, DMSO solution, and kits for MDA and Annexin–V–FITC apoptosis detection were bought from Solaibao Biotechnology Co., Ltd. Kits to determine the activity of SOD, GSH-Px, CAT, LDH, ALT and bicinchoninic acid (BCA) were purchased from Nanjing Jiancheng Institute of Biotechnology, China. Antibodies against caspase-3 and caspase-were purchased from Lianke Biotechnology Co. (Hangzhou, China). The trypan blue dye solution was obtained from Shanghai Yuanye Biotechnology Co. (Shanghai, China).

### 3.2. Extraction and Purification of MLF

The extraction, purification, and chemical characterization of MLF was performed and published in our previous study [22]. The chemical composition of MLF extract has been listed in Table 1 from the result of Zheng et al. 2022, [22]. Mulberry leaves were obtained from Shanxi, China. The dried plants were ground and filtered through a 60-mesh sieve to obtain a homogeneous sample powder, which was then placed in a desiccator for use. The extract of mulberry leaves was extracted by ultrasonic-assisted enzyme extraction (UAEE) with 70% ethanol (50:1, water-to-object ratio, mL/g) at pH 5 at 60 °C, and hydrolyzed by adding 5% cellulase and pectinase (1:3, g/g) for 40 min, and then processed by ultrasonic waves (Kunshan Ultrasonic Instrument Co., Ltd., Kunshan, China) at a power of 420 W for 15 min, and the mixture was filtered and concentrated to remove ethanol, and then diluted to 1 mg/mL. The solution was diluted to 1 mg/mL and purified by D101 macroporous resin to obtain MLF.

### 3.3. Cell Culture and Treatment

HepG2 cells were obtained from the Shanghai Cell Bank of the Chinese Academy of Sciences and cultured routinely in DMEM with 10% FBS, 1% double antibody (100 U/mL penicillin and 100 U/mL streptomycin) in a humidified incubator (5% CO_2_, 37 °C). HepG2 cells can be passaged when they have grown to about 80% of the bottom of the flask.

### 3.4. Cell Viability Assay

HepG2 cell viability was determined in a previous study and modified [15,16,23]. The cytotoxicity of H_2_O_2_ and MLF in HepG2 cells was determined by CCK–8 method. A volume of 100 μL of cell suspension was absorbed and inoculated onto 96-well plates at a density of 1 × 10^4^ cells/well, and the cells were divided into control and experimental groups and incubated in an carbon dioxide incubator (BB150, Thermo Fisher Scientific Inc., Waltham, MA, USA) at 5% CO_2_, 37 °C for 24 h. After removing the medium, HepG2 cells were incubated with 100 μL of MLF containing different concentrations (15.625, 62.5, 125, 250, 500, 1000 μg/mL) for 24 h (or H_2_O_2_ (600, 650, 700, 750, 800, 850, 900 μM) for 5 h. Finally, a medium containing 10% CCK–8 solution was added to each well for 1 h. OD values were measured at 450 nm using a full-wavelength enzyme labeler (Epoch, BioTek Instruments, Inc., Winooski, VT, USA).

(1) $$Cell\;viability\left(\%\right)=\frac{OD\;control\;groups-OD\;experimental\;groups}{OD\;control\;groups}\times 100$$

### 3.5. Influence of MLF on the Survival Rate of H_2_O_2_–Mediated Oxidative Injured HepG2 Cells

After HepG2 culture for 24 h, the cell suspension was removed and inoculated onto 96–well plates at a density of 1 × 10^4^ cells/well, and the cells were divided into the control group, the MLF experimental group and the damage group. They were prepared as follows: the MLF experimental group was incubated with 100 μL MLF (62.5, 125, 250, 500 μg/mL) for 24 h, and then 100 μL H_2_O_2_ (750 μM) was added to each well and incubated for 5 h; the damage group was incubated with 100 μL DMEM medium for 24 h, and then 100 μL H_2_O_2_ (750 μM) for 5 h; the control group was incubated with 100 μL of medium for 29 h. The survival rate of HepG2 cells was determined according to the method in Section 3.4.

### 3.6. Determination of Apoptosis Rate of HepG2 Cells

The apoptosis rate of HepG2 cells was referenced to the method of Wang et al. [20] with slight modifications. HepG2 cells were collected from the control, MLF-protected and damage groups in 3.5 Summary methodology. The apoptosis rate of HepG2 cells was detected by flow cytometry (CyoFLEX S, Beckman Coulter, Inc., Brea, CA, USA) according to the Annexin V–FITC/PI kit instructions.

### 3.7. Hoechst 33342 and Trypan Blue Staining Test

HepG2 cells from the control, MLF-protected and damage groups in 3.5 Summary methodology were collected according to the instructions of the Hoechst 33342 reagent and trypan blue reagent, respectively. The cells were incubated in the dark at room temperature analyzed by fluorescence inverted microscopy (LW300LFT, Shanghai Measurement Dimension Optoelectronic Technology Co., Ltd., Shanghai, China) according to the instructions of the Hoechst 33342 kit and trypan blue reagent, respectively.

### 3.8. Determination of Intracellular ROS Level

HepG2 cells were collected from the control, MLF–protected and damage groups in 3.5 Summary methodology The cells were centrifuged at 1000× *g* for 5 min and the cell deposits were collected. The fluorescence value of the control group was used as a control, and the ROS level of HepG2 was measured according to the instructions of the ROS assay kit.

### 3.9. Determination of Biochemical Parameters in HepG2 Cells

Collect the cell deposits according to the method in 3.5 Summary methodology. The treated cells were washed with pre-cooled PBS, lysed using high-efficiency RIPA lysis solution, collected in centrifuge tubes, centrifuged for 10 min, and the supernatant was collected for protein concentration detection using the BCA kit. MDA content, ALT and LDH leakage and SOD, GSH-Px and CAT activities were measured according to the kit instructions.

### 3.10. Determination of Mitochondrial Membrane Potential in HepG2 Cells

Collect the HepG2 cells after H_2_O_2_ incubation in 3.5 Summary methodology, aspirate the culture fluid, wash the cells once with PBS buffer solution according to the instructions of the mitochondrial membrane potential assay kit, briefly, then add 1 mL of cell culture medium, add 1 mL of JC-1 staining working solution and mix thoroughly, and place in a cell culture incubator for 20 min at 37 °C. During the incubation, of 4 mL per 1 mL of JC–1 staining buffer (5×) was added to 4 mL of distilled water to prepare the appropriate amount of JC–1 staining buffer (1×), and placed in an ice bath. After incubation at 37 °C, the supernatant was aspirated and washed twice with JC–1 staining buffer (1×), and finally, 2 mL of cell culture medium was added, observed under a fluorescent inverted microscope, and photographed.

### 3.11. Determination of Apoptosis Proteins Activities in HepG2 Cells

HepG2 cells in 3.5 Summary methodology were collected, the treated cells were washed with pre-cooled PBS, the cells were lysed using high-efficiency RIPA lysis solution, collected in centrifuge tubes, centrifuged for 10 min, and the supernatant was collected for relevant assays. Caspase-3 and Caspase-9 activities were detected using the Caspase kit according to the instructions.

### 3.12. Scanning Electron Microscope (SEM) Observation

Cell precipitates were collected according to the method in 3.5 Summary methodology. After gold spraying, the blank control group, the MLF-protected group (500 μg/mL MLF) and the damage group were scanned under a scanning electron microscope (Mira, Tescan, Czech) to observe the changes in cell morphology and structure.

### 3.13. Statistical Analysis

All experiments were triplicated. Results are expressed as the mean standard deviation. SPSS version 17.0 (SPSS17.0, Chicago, IL, USA) GraphPad (Prism 8, San Diego, CA, USA), and one-way ANOVA was used to analyze the significance of the differences in the results with a significance level of *p* < 0.05.

## 4. Discussion

Oxidative stress, a key factor in various diseases and ageing, has prompted research into natural compounds with antioxidant properties. Flavonoids, abundant in plant-based foods, are of particular interest due to their potent antioxidant activities. Coenzyme Q10 (CoQ10) has been found to protect CHO-K1 cells from oxidative stress induced by arsenic and zinc, highlighting its potential as a therapeutic agent against toxin-induced damage [24]. Similarly, *Pseudobombax parvifolium* hydroalcoholic bark extract (EBHE) shows significant antioxidant properties without cytotoxic or mutagenic effects, suggesting its potential for traditional medicine [25]. Green tea extract (GTE) and epigallocatechin-3-gallate (EGCG) exhibit promising antithrombotic, antitumor, and antiangiogenic activities, making them candidates for cancer treatment [26]. *Licania rigida* leaf extract (AELr) demonstrates therapeutic benefits with no observed toxicity or genotoxicity, while black mulberry extracts and seed oil show significant antioxidant and hepatoprotective effects [27,28,29].

Our study investigated the hepatoprotective properties of MLF against oxidative stress-induced damage in HepG2 cells. MLF demonstrated a concentration-dependent inhibition of oxidative damage induced by H_2_O_2_, with higher concentrations showing enhanced protective effects. Treatment with MLF reduced ROS levels, restored mitochondrial membrane potential, and decreased abnormal lactate dehydrogenase and alanine transaminase leakage induced by H_2_O_2_. MLF also modulated the activity of antioxidant enzymes, enhancing cellular defense mechanisms against ROS-induced damage. Flow cytometry analysis revealed that MLF attenuated ROS-induced apoptosis by regulating the expression of apoptosis-related proteins. These findings suggest that MLF holds promise as a natural hepatoprotective agent against oxidative stress, providing valuable insights into the potential use of flavonoid-rich functional foods as a preventive strategy against oxidative damage-associated diseases and ageing [30].

## 5. Conclusions

We focused on the protective capabilities of MLF against oxidative stress induced by hydrogen peroxide (H_2_O_2_) in HepG2 cells. Our study aimed to validate MLF’s potential as a hepatoprotective agent. Through our analysis, we found that MLF demonstrated a dose-dependent protective effect, notably reducing reactive oxygen species (ROS) levels and restoring mitochondrial function. Moreover, MLF exhibited the ability to regulate antioxidant enzymes and mitigate DNA damage. MLF’s promising role in mitigating oxidative stress-related damage in HepG2 cells underscores the significance of flavonoid-rich functional foods in promoting cellular health.

## Figures and Tables

**Table 1:** The Chemical composition of MLF extract.

Number	Compound	Formula (M)	Calculated Conc. (ng/mL)
1	Rutoside	C_27_H_30_O16	125,000
2	Hyperoside	C_21_H_20_O_12_	68,300
3	Catechin	C_15_H_14_O_6_	163
4	Myricitrin	C_21_H_20_O_12_	44,500
5	Isoquercitrin	C_21_H_20_O_12_	43.1
6	Kaempferol 3-rutinoside	C_27_H_30_O_15_	15,700
7	Paeoniflorin	C_23_H_28_O_11_	3570
8	Epicatechin	C_15_H_14_O_6_	64.2
9	Taxifolin	C_15_H_12_O_7_	518
10	Quercetin	C_15_H_10_O_7_	886
11	Luteolin	C_15_H_10_O_6_	95.6
12	Morin	C_15_H_10_O_7_	698
13	Astragalin	C_21_H_20_O_11_	16,700
14	Quercitrin	C_21_H_20_O_11_	18,600
15	Isorhamnetin-3-O-glucoside	C_22_H_22_O_12_	3.65
16	Curculigoside	C_22_H_26_O_11_	23.0
17	Hesperidin	C_28_H_34_O_15_	180,000
18	Cynaroside	C_21_H_20_O_11_	16,200
19	Vitexin	C_21_H_20_O_10_	4990
20	Kaempferol	C_15_H_10_O_6_	209
21	Guaiaverin	C_20_H_18_O_11_	12,300
22	Licochalcone-A	C_21_H_22_O_4_	15.2
23	Ginsenoside Rg2	C_42_H_72_O_13_	116
24	Ginsenoside C-K	C_36_H_62_O_8_	211
25	Ginsenoside Rc	C_53_H_90_O_22_	230
26	Ginsenoside Rg1	C_42_H_72_O_14_	1150
27	Ginsenoside Rf	C_42_H_72_O_14_	4.54
28	Ginsenoside F1	C_36_H_62_O_9_	3140
29	Ginsenoside Rb2	C_53_H_90_O_22_	110
30	Ginsenoside Rd	C_48_H_82_O_18_	71.2
31	Ginsenoside Re	C_48_H_82_O_18_	284
32	Hesperetin	C_16_H_14_O_6_	1190
33	Polydatin	C_20_H_22_O_8_	318
34	Cyanidin 3-O-glucoside	C_21_H_21_ClO_11_	64,800
35	Cyanidin 3-O-rutinoside	C_27_H_31_O_15_	39,600
36	Isorhamnetin-3-O-glucoside	C_22_H_22_O_12_	191
37	Naringenin	C_15_H_12_O_5_	341
38	Apigenin 7-glucoside	C_21_H_20_O_10_	6680
39	Cyanidin	C_21_H_28_O_8_	19,500

## Data Availability

The data used to support the findings of this study are available from the corresponding author upon request.
